# Food Supply Chain Stakeholders’ Perspectives on Sharing Information to Detect and Prevent Food Integrity Issues

**DOI:** 10.3390/foods8060225

**Published:** 2019-06-25

**Authors:** Fien Minnens, Niels Lucas Luijckx, Wim Verbeke

**Affiliations:** 1 Department of Agricultural Economics, Faculty of Bioscience Engineering, Ghent University, Coupure Links 653, 9000 Ghent, Belgium; wim.verbeke@ugent.be; 2 TNO, Utrechtseweg 48, 3704 Zeist, The Netherlands; niels.lucasluijckx@tno.nl

**Keywords:** food integrity, transparency, food supply chain, information sharing, stakeholder, food fraud

## Abstract

One of the biggest challenges facing the food industry is assuring food integrity. Dealing with complex food integrity issues requires a multi-dimensional approach. Preventive actions and early reactive responses are key for the food supply chain. Information sharing could facilitate the detection and prevention of food integrity issues. This study investigates attitudes towards a food integrity information sharing system (FI-ISS) among stakeholders in the European food supply chain. Insights into stakeholders’ interest in participating and their conditions for joining an FI-ISS are assessed. The stakeholder consultation consisted of three rounds. During the first round, a total of 143 food industry stakeholders—covering all major food sectors susceptible to food integrity issues—participated in an online quantitative survey between November 2017 and February 2018. The second round, an online qualitative feedback survey in which the findings were presented, received feedback from 61 stakeholders from the food industry, food safety authorities and the science community. Finally, 37 stakeholders discussed the results in further detail during an interactive workshop in May 2018. Three distinct groups of industry stakeholders were identified based on reported frequency of occurrence and likelihood of detecting food integrity issues. Food industry stakeholders strongly support the concept of an FI-ISS, with an attitude score of 4.49 (standard deviation (S.D.) = 0.57) on a 5-point scale, and their willingness to participate is accordingly high (81%). Consensus exists regarding the advantages an FI-ISS can yield towards detection and prevention. A stakeholder’s perception of the advantages was identified as a predictor of their intention to join an FI-ISS, while their perception of the disadvantages and the perceived risk of food integrity issues were not. Medium-sized companies perceive the current detection of food integrity issues as less likely compared to smaller and large companies. Interestingly, medium-sized companies also have lower intentions to join an FI-ISS. Four key success factors for an FI-ISS are defined, more specifically with regards to (1) the actors to be involved in a system, (2) the information to be shared, (3) the third party to manage the FI-ISS and (4) the role of food safety authorities. Reactions diverged concerning the required level of transparency, the type of data that stakeholders might be willing to share in an FI-ISS and the role authorities can have within an FI-ISS.

## 1. Introduction

Integrity challenges along the food supply chain have received increasing attention by food safety authorities, industry and media in recent years. Adverse impacts of the adulteration of food products extend far beyond direct effects on the food industry itself. Indirect effects relate, amongst others, to the loss of public and consumer confidence in food production, the food industry, and food safety and quality [1,2]; international trade distortions and disputes [3]; and effects on food policies and consumer politics [4]. Following the BSE (Bovine Spongiform Encephalopathy) crisis at the end of the 1990s, Verbeke and Ward [5] showed that negative media coverage largely outweighs similar amounts of positive coverage aimed at reassuring the market and consumers. Therefore, eliminating the grounds for negative press, such as by following food integrity issues, emerges as a key attention point for the food supply chain.

A variety of measures are being developed and applied to safeguard food safety and quality, and to detect and prevent food integrity issues by different actors, both technical and organizational. Ellis, Muhamadali, Haughey, Elliott, and Goodacre [6] stressed that the ever-expanding portfolio of analytical methods, techniques and technologies in food control and future pervasive and predictive computation will together take on the role of a technology-based capable guardian for food systems. Simultaneously, more than ever before, experts recognize that food integrity is a challenge that requires a joint strategy and coordinated efforts involving all stakeholders, and that a strengthening of the collaboration between industry and governments is necessary [7]. The development of an integrated private–public strategy requires clearly defined roles for each participating stakeholder and clarity and shared agreement on the specific purpose [8].

The Elliott review following the 2013 horsemeat incident introduced eight pillars of food integrity: consumers first, zero tolerance, intelligence gathering, laboratory services, audits, government support, leadership, and crisis management [9]. The recommendations that are formulated for these eight pillars refer multiple times to the need for data, information and intelligence sharing between stakeholders: “*There needs to be a shared focus by Government and industry on intelligence gathering and sharing. The Government should work with the Food Standards Agency (to lead for the Government) and regulators to collect, analyze and distribute information and intelligence; and work with the industry to help it establish its own ‘safe haven’ to collect, collate, analyze and disseminate information and intelligence.”* [9] (p. 7). Following the horsemeat incident, several actions were taken, and new initiatives were set up. For example, in the United Kingdom, the incident led to the establishment of the Food Industry Intelligence Network (FIIN), and on a European level it led to the Food Fraud Network (FFN), both aiming at the type of intelligence gathering that the Elliott review recommended [7].

Information and data that could be relevant to identify potential issues of integrity in food supply chains are often initially only available to industry experts operating at a specific level of the agro-food supply chain. Ideally, this information and data would be shared, integrated and analyzed, in order to help reveal issues faster and more accurately, and to help prevent them. Although the integration of food integrity data and information covering the whole food supply chain in one digital system seems futuristic, the digital data revolution and developments in artificial intelligence are transforming many economic sectors already, and the food sector is often named as one that might benefit substantially from a similar transition too [10,11]. Several studies have explored ways to implement such a holistic approach to identify, detect or prevent food integrity issues. For example, Bayesian network models entail the potential for predicting increased likelihoods of the occurrence of incidents [12,13]. The potential adoption of the Internet of Things (IoT) in the agro-food supply chain seems promising but several challenges and constraints were identified by Brewster et al. [14]. Decentralized traceability systems such as Blockchain structures are gaining interest and could provide solutions to these constraints [15,16].

While the development of analytical methods, technologies, systems, and infrastructures is gaining momentum, questions relating to stakeholder acceptance, intentions, and their willingness to adopt and participate remain largely unaddressed thus far. It is important to assess stakeholders’ attitudes towards data and information sharing and how information sharing systems will be received, with the goal of detecting and preventing food integrity issues. The aim of this study was to investigate food supply chain stakeholders’ attitudes and intentions towards a food integrity data and information sharing system (further referred to as an FI-ISS). The consultation of stakeholders focused on three objectives which were addressed in three consecutive rounds of data collection. Firstly, we aimed to analyze how food industry stakeholders receive the idea of an FI-ISS, which preconditions they consider important and what explains their intention to join an FI-ISS or not. The second objective of this study was to determine key success factors for the successful development and adoption of an FI-ISS. Lastly, the study explored in further detail the meaning of the defined key success factors, specific sensitivities facing the introduction of an FI-ISS and the origins of eventual contentious points.

## 2. Materials and Methods

This study combined exploratory and descriptive conclusive research methods and consisted of three consecutive rounds of data collection in which different stakeholders active in the European food supply chain were consulted. The first round (November 2017–February 2018) focused on food industry actors (*n* = 143), while, during the second (March–April 2018) (*n* = 61) and third rounds (May 2018) (*n* = 37), the target group was broadened beyond food industry stakeholders alone. The type of stakeholders that participated and their distribution across stakeholder groups are presented in Table 1.

The first round consisted of an online survey using a quantitative questionnaire, with the aim of identifying food industry actors’ attitudes towards, views on, intentions to join and preconditions for the uptake of an FI-ISS. The questionnaire was web-programmed in Qualtrics and covered industry stakeholders’ perceptions about food integrity issues, their attitudes towards the concept of sharing information to detect and prevent food integrity issues, and specific attitudes and intentions towards an FI-ISS. In order to inform participants and ensure consistent framing of the potential FI-ISS, participants were exposed to a 150 second explanation video that explained the main characteristics of an FI-ISS [17].

After watching the video, 143 stakeholders started the survey, of which 111 continued until the end of the survey. All descriptive results are presented for each question using all valid answers, i.e., including all data for those participants that have answered that specific question. The participants are stakeholders with diverse relevant responsibilities in their organization, with 87% of them being employed in quality or R&D management. The rest of the participants (13%) had responsibilities such as purchase management, general management, sales management or consultancy. Their businesses or organizations are active on different levels of the food supply chain such as processing (68%), primary production (25%), retail (21%) and services to the food industry (10%) such as software or packaging. Two-thirds are active in international trade. The food commodities they are actively working with cover the food commodities most vulnerable to food integrity issues [18]. All of the 111 participants that completed the survey from start to finish reported dealing with one or more of the most vulnerable commodities, namely, organic food (39%), milk (35%), grains (31%), spices (28%), fish (23%), olive oil (21%), honey and syrups (21%), fruit juices (19%), coffee and tea (17%), wine (10%) and meat (9%). This could be due to the fact that the topic of the survey, being food integrity issues, appeals more to those working in more vulnerable sectors, which might have increased their interest and motivation to participate in the study. Therefore, when interpreting these results, it is important to take the background of participants and possible self-selection bias into account.

First, participants were asked to report the frequency of occurrence of food integrity issues and their likelihood of detection within their own organization. Both items were measured on a 6-point categorical scale. Additionally, the perceived risk of food integrity issues was measured using three items on a 5-point Likert scale. Second, participants’ attitudes towards information sharing with the aim of tackling food integrity issues were assessed using three items (negative–positive, uninteresting–interesting, unimportant–important) on a 5-point bipolar interval scale. In a similar vein, perceived usefulness of information sharing was measured using three items (useless–useful; irrelevant–relevant; unnecessary–necessary). Three items on a 5-point Likert scale were used to measure how stakeholders perceive the risk of food integrity issues including “My company is very concerned about becoming a victim of food fraud”, “Food integrity issues are a growing problem in our sector” and “Food integrity issues are one of the main risks our company faces”. Third, participants were exposed to a series of potential advantages (*n* = 8) and disadvantages (*n* = 6) of information sharing; conditions for joining (*n* = 16) an FI-ISS; intention to join an FI-ISS (*n* = 7); third parties (*n* = 9) that could manage the FI-ISS; types of data (*n* = 9) that could be shared; and minimal output of the FI-ISS (*n* = 7), and were asked to indicate their degree of agreement using 5-point Likert scales. The selection of possible advantages, disadvantages, conditions for joining, third parties, the output of the system and types of data was based on literature review and expertise within the research team. For each set of items, participants were provided with the option to add additional items if they felt crucial items were missing. The construct scores for perceived advantages, perceived disadvantages, perceived risk and intention to join an FI-ISS were obtained by aggregating the items, leading to mean scores. Internal consistency was assessed with Cronbach’s α values. Finally, characteristics of the organization were recorded such as type of activity, food commodities or product groups covered, geographical scope (regional, national, pan-EU, global), and size (micro, small, medium-sized, large).

**Figure 1:**
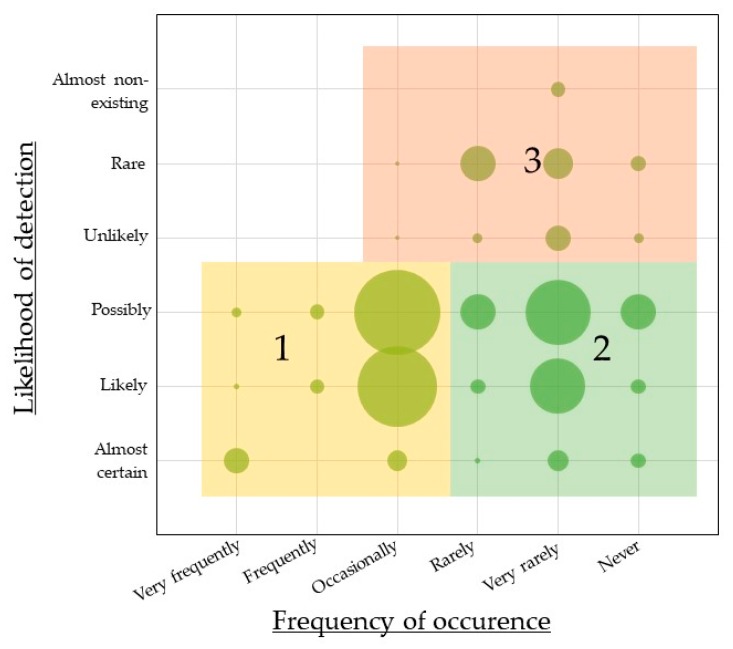
Bubble chart mapping food industry actors’ perceptions of the occurrence of food integrity issues and the likelihood of detecting issues within their companies, identifying three clusters (*n* = 133, bubble sizes refer to *n*, 1 = Cluster 1, 2 = Cluster 2, 3 = Cluster 3).

**Figure 2:**
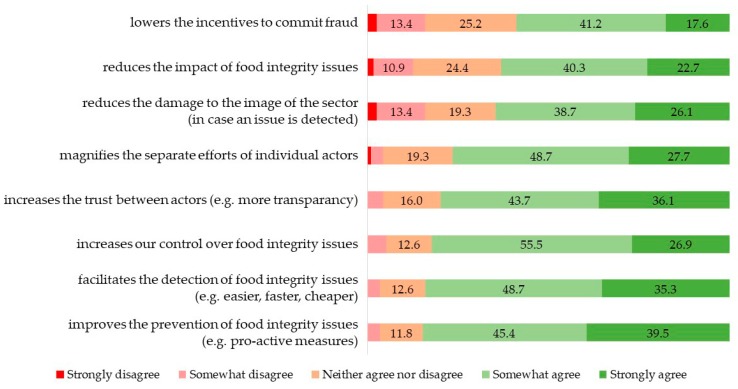
Perceived advantages of information sharing to prevent and detect food integrity issues according to food industry actors (*n* = 119, %).

The results of the first round were summarized and presented to the participants of the second round with summarizing text and bar charts. The stakeholders involved in the second round were thus exposed to the insights obtained from round 1 and invited to provide feedback and additional comments with the aim of identifying key success factors for an FI-ISS. The survey in the second round consisted of five sections. It assessed perception of current food integrity issues; the potential of information sharing; suitable trusted third parties; types of data to share; and initiatives for setting up an FI-ISS. A combination of closed-ended and open-ended questions was used, allowing the collection of both quantitative data and qualitative insight. As a graphical aid, a selection of the bar charts (as shown in the results section of this paper) were presented to the participants of round 2 (Figure 1 and Figure 2).

The third round of data collection consisted of an interactive workshop with stakeholders and experts involved in the food supply chain. The results of the previous rounds were shared with the participants by means of a plenary presentation, after which these were discussed in four parallel working group sessions with a moderator and following a discussion guide. Each working group provided feedback and conclusions, which were discussed in plenary. This third-round interactive workshop envisaged providing a better understanding of the meaning of different views and perspectives on the future application of an FI-ISS.

In the interest of gathering unrestrained answers from food industry stakeholders, an anonymous approach was adopted for both data collection rounds involving online surveys. Before starting the survey, participants were informed about the context and purpose of the study, and researcher’s contact details were provided. After completing the surveys, participants were invited to subscribe in a separate form (not linked to their responses) to receive feedback and an invitation for the next rounds. As such, the survey responses were never linked to personal identifiers and not linked between two rounds. All information was stored and processed in a non-identifiable format and reported in aggregated form only. Both surveys were distributed through an online link to a Qualtrics webpage. To reach a wide range of potential participants, multiple channels were contacted, and several federations and organizations agreed to share the survey links and workshop invitations within their professional networks, including the national federations of the Belgian, French and Dutch food industries (Fevia, ANIA, FNLI), specialized press such as Food Quality News and through the Food Integrity Network. 

A challenge in this study was avoiding the dropout of participants during the consecutive rounds of the study. The questionnaire was concise and clear to limit the number of participants dropping out during the survey and to avoid survey fatigue. After the first round, it was explained to participants that they would receive the aggregate findings of the first round when staying involved in the study, with the aim of motivating them to take part in the next rounds. However, the possible efforts to avoid dropout were limited as a result of the anonymization, which made it impossible to send personalized reminders to participants. Statistical analyses were performed with SPSS Statistics 23.0 (IBM SPSS, Armonk, NY, USA), and qualitative responses to open-ended questions were analyzed with QSR International’s NVivo 11 qualitative data analysis software (Melbourne, Australia). Cronbach’s α values were computed to assess the internal consistency of measurement scales. Quantitative data processing and analysis included descriptive (frequency distributions), bivariate (correlations, chi-square tests, *t*-tests, and ANOVA) and regression analyses. Stepwise linear regression was adopted to identify predictors of the intention to join an FI-ISS. The Breusch–Pagan test was conducted to verify whether the assumption of homoscedasticity was satisfied. Qualitative data from open-ended questions were coded into categories for interpretation purposes.

## 3. Results and Discussion

### 3.1. Quantitative Survey with Food Industry Actors

#### 3.1.1. Perceived Occurrence and Likelihood of Detection of Food Integrity Issues

A total of 143 industry stakeholders completed the questions about the occurrence of food integrity issues and the likelihood of the detection of issues within their own organization. The participants were categorized into clusters by combining these two variables. The chart in Figure 1 shows the frequency distribution of the reported occurrences and likelihoods of detection on the x- and y-axes, respectively, while the diameter of the circles represents the number of participants with that specific combination of responses. For example, an almost equal number of participants reported an occasional frequency of occurrence combined with the expected detection of the issue being classified as “likely” (*n* = 16) or “possibly” (*n* = 17). 

Figure 1 also illustrates the three different clusters that were identified. Cluster 1 (*n* = 51) consists of food industry actors who perceive both the frequency of occurrence of issues and the likelihood of detecting issues as high. Cluster 2 (*n* = 52) is a group of actors who consider the frequency of food integrity issues to be low, and the likelihood that they would detect issues to be high. The smaller cluster 3 (*n* = 30) contains industry actors who perceive both the occurrence and likelihood of the detection of food integrity issues as low. Participants who indicated that they were unaware of the frequency of occurrence of food integrity issues within their organization (*n* = 10) were not included. The stakeholders in the three clusters differ significantly in terms of company sizes (Chi-square = 17.5; *p* = 0.001). Compared to the distribution in the sample, Cluster 3 is overrepresented by stakeholders from medium-sized enterprises (47.8%), while these are underrepresented in cluster 1 (5.1%). Cluster 2 consists of a balanced mix of stakeholders from different company sizes. This finding suggests that medium-sized companies perceive the likelihood of detecting food integrity issues as lower compared to smaller or larger companies. On one hand, smaller companies might have a better view and control over issues because of the smaller scale of the company. Larger companies, on the other hand, might feel more in control because of experience, economies of scales, or eventual measures they may have already taken to detect food integrity issues. There are no significant differences between the clusters in terms of business characteristics or attitudes towards information sharing (all *p* > 0.05). However, there is a difference in the perceived risk of food integrity issues. The mean perceived risk score (Cronbach’s α = 0.67) is 3.44 (standard deviation (S.D.) = 0.89) for the total sample. Stakeholders in cluster 1 perceive the risk as significantly higher (µ = 3.70, S.D. = 0.90) compared to those in cluster 2 (µ = 3.26, S.D. = 0.87) (*p* = 0.045).

#### 3.1.2. General Attitude and Perceived Advantages and Disadvantages of Information Sharing

Food industry stakeholders’ attitudes towards information sharing with the aim of tackling food integrity issues were measured on 5-point bipolar interval scales. The mean attitude score across the three items (Cronbach’s α = 0.85) was 4.49 (*n* = 119, S.D. = 0.57), which indicates that, in general, participants have a very positive attitude towards information sharing to detect and prevent food integrity issues. To measure participants’ perception of the usefulness of information sharing, they were asked to rate three items on a 5-point bipolar interval scale. Cronbach’s α for the three items was 0.81, indicating very good internal consistency reliability. The three item scores were aggregated and averaged to obtain an overall attitude score. The mean attitude score was 4.52 (*n* = 119, S.D. = 0.54), which indicates that, in general, participants perceive information sharing as very useful to detect and prevent food integrity issues.

In competitive environments, information sharing has advantages and disadvantages, depending on the aim and context for the different actors in the food supply chain and for the sector as a whole. First, participants were asked to score eight statements regarding potential advantages of information sharing. Figure 2 shows a strong consensus among stakeholders about most of the potential advantages, with less than 5% of participants disagreeing. However, disagreement is higher (more than 10%) for ‘reduces the damage to the image of the sector’, ‘reduces the impact of food integrity issues’ and ‘lowers incentives to commit fraud’. The lack of consensus on these advantages implies that they are not obvious for all stakeholders, which suggests that possible doubts about the impact on the sector will need to be addressed when developing an FI-ISS. Discussions with stakeholders in the following rounds showed that there is a concern about the response, e.g., overreaction when issues are detected in an FI-ISS. The fear of overreaction might explain these doubts about the advantages for the sector. Although it is difficult to predict the impact of the adoption of an FI-ISS on the image of the food sector, Charlebois and Haratifar [19] concluded that the introduction of a food traceability system in the organic dairy sector was perceived as valuable by dairy consumers and suggested that information sharing entailed the potential to increase the market share of dairy products. To measure stakeholders’ overall perception of the advantages, a mean aggregated score was calculated of the eight items (Cronbach’s α = 0.770). The mean of stakeholders’ perceived advantages is 3.96 (S.D. = 0.55).

Sharing information within a food supply chain also might entail drawbacks for the stakeholders involved. The feasibility of an FI-ISS will depend strongly on its potential to avoid or tackle these pitfalls. Mapping the possible pitfalls or doubts of stakeholders enables a better definition of the requirements for an FI-ISS. Six statements were used regarding potential disadvantages of information sharing. The overall perception of the disadvantages of joining an FI-ISS was measured as a mean aggregated score of the six items (Cronbach’s α = 0.748), which amounted to 3.25 (S.D. = 0.64). No significant differences in perceived disadvantages were found between different company sizes, geographical scopes or clusters. Stakeholders’ perceptions of disadvantages of an FI-ISS are negatively correlated with how they perceive the advantages (*r* = −0.403, *p* < 0.001).

**Figure 3:**
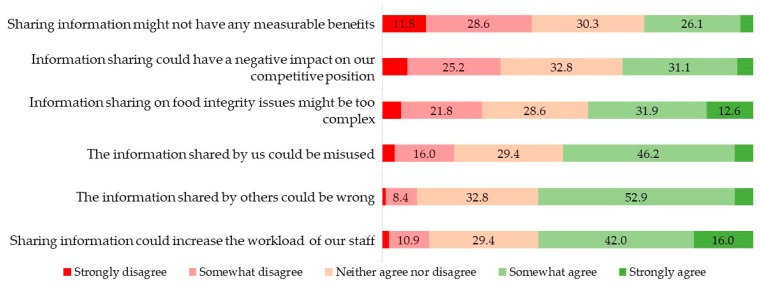
Perceived disadvantages of information sharing to prevent and detect food integrity issues according to food industry actors (*n* = 119, %).

Figure 3 shows that there is less consensus about disadvantages of information sharing compared to the level of agreement on its advantages. One of the main perceived disadvantages is that information sharing might increase the workload of staff, with 58.0% of participants confirming that they consider this a potential disadvantage. Hence, during the development of an FI-ISS, user-friendliness and the avoidance of too much additional workload should be taken into account. In a U.S. study where stakeholders evaluated options for improving product traceability, it was emphasized that the end solution should create net positive value for consumers and all companies in the food supply chain [20]. A potential FI-ISS has to be able to convince people that the overall benefits for food businesses will offset the costs. Over half of the stakeholders also showed distrust towards other actors, with the concern that they might share the wrong information or misuse the FI-ISS. In a similar vein, about one third of participants agreed that information sharing could have a negative impact on their competitive position. Interestingly, another third disagreed with this statement, i.e., they do not expect disadvantages in terms of competitive positions. In recent years, several experts have expressed their belief that food integrity should be considered a pre-competitive issue in a similar vein as food safety, i.e., a non-negotiable condition for market access. The Elliot Review urged the food industry to include food crime prevention in companies’ corporate social responsibility policy and acknowledged that it can deliver commercial benefits [9].

#### 3.1.3. Stakeholders’ Conditions for Joining an FI-ISS

There is a clear consensus on several conditions that an FI-ISS needs to meet. First and foremost, industry actors agreed that they would need access to how the data and information that is shared will be handled, with the necessary protocols and procedures in place. Consequently, an important aspect is the confidentiality and encryption of the data and information, and also the anonymity of companies involved. The results show that industry actors consider this to be one of the main prerequisites; in other words, they would not join if their identity is not protected. There is also consensus on the importance of well-defined roles and rights of the trusted third party that manages the FI-ISS. Industry actors strongly agreed that data from authorities, research institutes, and NGOs should be incorporated in an information sharing system. Earlier research on the use of big data to identify emerging risks using Bayesian Network Modelling showed that several data sources can be used in predictive models [13]. For example, Bouzembrak et al. [21] showed how media reports on food fraud can be a useful input source for prediction systems. The participation of a sufficient number of actors in the sector in an FI-ISS is also a precondition for 82.3% of participants in our survey.

#### 3.1.4. Intention and Its Determinants

Participants were asked about their likelihood to join an FI-ISS if their requirements were met. About 75% of participants reported that they would share all relevant information with an FI-ISS. Moreover, over 80% claimed that they would recommend an FI-ISS to their suppliers; 67% would recommend it to their customers, and 62% would recommend it to their competitors. The participants received a rather abstract description of the concept an FI-ISS, without any mentioning of funding or cost. However, in the survey, almost one third (31.5%) of participants expressed that they would be willing to pay for access to an FI-ISS. 

The seven statements about the likelihood to join are considered a measure of the intention of stakeholders to join an FI-ISS. The aggregated score shows strong internal consistency (Cronbach’s α = 0.879). Stakeholders’ mean intention to join an FI-ISS is 3.81 (S.D. = 0.71). The intention to join an FI-ISS is significantly different for stakeholders from different company sizes. Medium-sized companies (µ = 3.47, S.D. = 0.71) have a significantly lower intention to join an FI-ISS compared to large companies (µ = 3.97, S.D. = 0.68). No significant differences in intentions were found in terms of other company characteristics such as geographical scope or cluster. On one hand, a positive and significant correlation was found between the intention to join an FI-ISS and the perceived advantages of an FI-ISS (*r* = 0.280, *p* = 0.003). On the other hand, a negative significant correlation was found between intention and perceived disadvantages (*r* = −0.208, *p* = 0.028). Although no significant correlation was found between a stakeholder’s perception of the risk of food integrity issues and their intention to join, the perceived risk is positively correlated with the perception of advantages (*r* = 0.293, *p* = 0.002).

To assess the joint impact of these factors and company size as predictors of intention to join an FI-ISS, a stepwise linear regression analysis was adopted. With each step, explanatory variables are evaluated based on their significance level (Table 2). For the categorical variable size of the company, the category ‘Micro and small’ was considered the baseline category and ‘Medium’ and ‘Large’ were coded as dummy variables for comparison. The variables of perceived advantages, perceived disadvantages and perceived risk were entered as possible explanatory variables. The Breusch–Pagan test indicated that the assumption of homoscedasticity is satisfied (*p* = 0.800).

Table 2 shows that perceived advantages and the size ‘medium’ were significant determinants of a stakeholder’s intention to join an FI-ISS. Perceived advantages or benefits were also positively related to traceability systems implementation and the readiness for the implementation of a halal assurance system, for example, among small and medium-sized enterprises (SMEs) in the study of Abd Rahman et al. [22]. The dummy “medium size” is significant in the model with a negative coefficient, confirming that medium company size lowers the intention to join an FI-ISS. This may suggest different things, e.g., that medium-sized companies experience a lower urgency of dealing with food integrity issues, eventually as a result of having been less confronted with observed food integrity issues or simply being less convinced that information sharing might provide a solution to food integrity issues. This potential explanation is hypothetical, and this empirical finding is of interest to be further studied in the future. Other variables such as perceived disadvantage and perceived risk were not significant.

#### 3.1.5. Suitability of Third Parties to Manage an FI-ISS

One of the main questions about organizing an FI-ISS is the choice of the trusted third party that will manage the system. A majority of 84.1% of participants agreed that a new organization established for this specific purpose would be the most suitable third party. This raises the question of whether the establishment of such a new organization can be realized in practice. Additionally, a food safety authority is also perceived to be a suitable third party by 74.3% of the industry stakeholders.

#### 3.1.6. Information and Communication 

An FI-ISS would gather information from different sources, analyze and manage it and produce an output of information which can be accessed by the members. For an FI-ISS to be effective, data and information need be shared by food businesses. There are three types of data which more than 70% of participants agreed could be shared within an FI-ISS: monitoring and surveillance data, analytical data on product content, and data on certifications. However, it is important to note that for analytical data, 11.7% did not agree that these could be shared. It might be interesting to further analyze why a substantial share of industry stakeholders are reluctant to share that type of information. This coincides with 12.6% disagreeing with the potential sharing of information on the sourcing of their raw materials and ingredients. Less than half of the participating stakeholders confirmed that they would be willing to share import or trade data at company level, data on volumes or transactional data. The reluctance to share such commercially sensitive information can be due to the fear of losing competitive advantage. In addition, in a recent stakeholder study on the acceptance of blockchain technologies in meat supply chains, interviews with government officials showed that they doubted the feasibility of complete transparency and traceability [23]. The authors suggested the introduction of legal obligations to ensure that all actors provide complete information. In a similar vein, Bhatt et al. [20] pointed out that striking a balance between voluntary and regulatory compliance will be critical in the improvement of traceability. 

An information sharing system can produce different types of output. The results of the survey show that stakeholders request clear protocols for action in case irregularities are detected. Additionally, stakeholders were very positive about the different possible outputs and agreed an FI-ISS should minimally produce a real-time searchable database, ad hoc alerts in case of irregularities and timely reports with filtered and analyzed information.

### 3.2. Insights from the Qualitative Survey 

**Figure 4:**
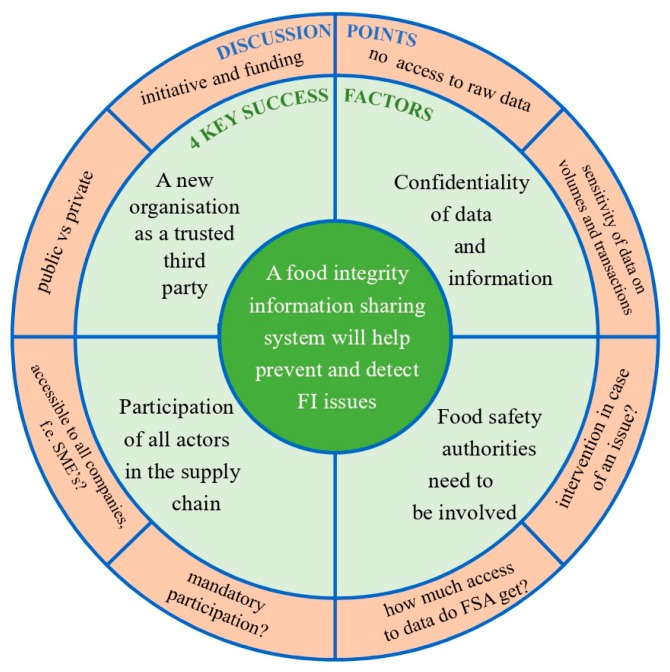
Four key success factors and associated discussion points for a food integrity information sharing system (FI-ISS). SME, small and medium-sized enterprise; FSA, food safety authorities.

A broader range of stakeholders were involved in the second round of the study to gather different perspectives. The distinction was made between food industry actors, including participants that identified themselves as part of the food industry or services to the food industry, and other types of actors (including researchers, food safety authorities and law enforcement). All participating stakeholders were asked to analyze the response of industry actors obtained in round 1 and give their opinion on those results in a survey with both closed-ended and open-ended questions. The qualitative analysis of the answers from round 2 resulted in the selection of four key success factors (KSFs) for an FI-ISS. The four KSFs are presented in Figure 4, and each of the four will be discussed in detail in this section.

#### 3.2.1. Potential of an FI-ISS

The positive reception of the concept of an FI-ISS was presented to the participants in round 2. The participants in round 2 answered an open-ended question on why they think an FI-ISS has the potential to detect and prevent issues with food integrity, in addition to the advantages/disadvantages already covered in round 1. The answers were coded into categories. The advantages that were mentioned most are: information sharing will raise awareness on food integrity issues (*n* = 12), and the detection of food integrity issues will be easier and faster (*n* = 11). A number of stakeholders also mentioned that information sharing will give more insights into weak spots in the food supply chain (*n* = 7). However, some of the stakeholders mentioned risks or barriers that they perceived, inhibiting the potential of information sharing. For example, some referred to the risk that fraudsters might abuse the information in an FI-ISS (*n* = 2), the doubt that some indicators are not reliable enough because existing methods are not robust (*n* = 2) and the risk of potential overconfidence when having an FI-ISS in place (*n* = 1).

#### 3.2.2. A New Organization as a Trusted Third Party

A first key success factor that became apparent from the results of the second round was the choice of a trusted third party. According to food industry actors in round 1, the two most suitable parties for organizing an FI-ISS were either a newly established organization or a food safety authority. Most of the stakeholders in round 2 confirmed that a newly established organization would be the most suitable trusted third party to manage an FI-ISS. Of the stakeholders (*n* = 7) who disagreed that a new organization should be the suitable third party, some mentioned the complexity of establishing and integrating another organization as the reason for their reluctance.

We also studied the idea of a new organization in more depth, by probing stakeholders’ opinions about the criteria that such an organization should fulfill. Analysis of the open-ended questions shows that there are diverging opinions on the type of new organization that should be in charge of an FI-ISS. Some stakeholders believe that a new organization would need to operate on an international level (*n* = 7), arguing that food markets are globalized. The majority of stakeholders indicated to prefer a new organization established at the EU level (*n* = 18); however, they rarely clarified the reason for this preference. A total of 21 participants stated that they prefer a non-profit organization. One of the stakeholders who preferred a private organization mentioned “*confidential information will be involved”* as the reason for this preference. Another stakeholder used the same argument for his/her preference for a public organization, stating *“usually these food integrity issues involve sensitive data covered by data protection legislation”.* Several stakeholders mentioned why they think public funding should be used to finance an FI-ISS, for example “*if there is no charge to the companies using it then it would be more effective—cost might be an issue to SMEs where finances are tight”.* Another argument related to the primary purpose of an FI-ISS, where a participant mentioned *“it must be public since its primary purpose must be to protect consumers”.* It is important to note also that four stakeholders expressed a preference for a public–private partnership, without further argumentation as to why.

#### 3.2.3. Confidentiality of Data and Information

Results from the first round showed there was a reluctance to share data on traded volumes and transactions. During the second round, stakeholders were asked for possible reasons for this reluctance. Analysis of the open-ended questions shows two main reasons. Firstly, most stakeholders mentioned the commercial sensitivity and the fear of competitive advantage issues. When setting up an information sharing system which aims to use the information on volumes and transactions, one needs to take these fears into account and foresee the necessary guarantees of anonymity and confidentiality of the information and data shared. Secondly, a few stakeholders also mention the fear of authorities having access to business information, more specifically finances and taxation authorities. Almost all stakeholders considered it necessary to anonymize data before they are seen by others. These results confirm that there is no consensus on how much access to data actors in a system should really have. A considerable group considered it not possible to give all actors access to raw data.

#### 3.2.4. Role of Food Safety Authorities (FSAs)

The majority of stakeholder participants in round 2 (78%) agreed that FSAs would be suitable as a trusted third party to manage an FI-ISS. FSAs exist on the national level but also on an international level, such as the EFSA (European Food Safety Authority). Stakeholders were asked for which of these they saw as having a role within an FI-ISS. The majority of participants indicated that this role can be for both (56%). Analysis of the comments made by stakeholders in the open-ended survey question which asked them to clarify their choice showed that eight stakeholders (consisting of one industry actor and seven non-industry actors) clearly expressed their preference for FSAs, considering them fit to manage an FI-ISS, and three reasons for this choice were expressed. Firstly, their already existing structure, network and expertise were mentioned, leading to a faster and less complex set-up of the system. Secondly, the already established trust among consumers was referred to. Thirdly, one stakeholder referred to the resources that FSAs have at their disposal. Another 12 stakeholders also mentioned that they see a role for FSAs and their expertise, but do not necessarily want them to be the third party that manages the FI-ISS, for example *“food safety authorities have an important role to play but are not equipped for this task. With the already existing systems they can play an important input role”.* Lastly, 13 stakeholders expressed the clear opinion that FSAs should not act as the third party managing an FI-ISS. A lack of trust among the industry was mentioned by four stakeholders, pointing at a lack of reliability and fear of sanctions. Furthermore, three stakeholders expressed their concern that FSAs should keep a certain distance from industry, for example, *“food safety authorities often have to maintain distance from industry, for fear of being assumed to be complicit with the food industry. Its action as a third party may prevent it from working appropriately*”. Another argument expressed by a few stakeholders is the difference between food safety issues and food integrity issues, and their worry that FSAs are not suitable to deal with the economic aspects of food integrity.

#### 3.2.5. Participation of All Actors in the Food Supply Chain

The results from the first round showed that food industry actors find the participation of all actors a key success factor for an FI-ISS. In the second round, the survey probed for participants’ opinions about the participation of consumer organizations and retailers. A number of stakeholders (*n* = 10) stated that they do not see a role for consumer or retail organizations, mentioning a few key arguments. Mostly, the commercial interest of retailers was an issue of concern. Stakeholders also mentioned lack of experience in dealing with food integrity issues, bad reputation and the fear that they would overreact to issues, for both retailers and consumer organizations. However, some stakeholders want a less active but rather advisory role for consumer organizations and retailers, for example, by being a member of the board of the new organization. Lastly, two stakeholders also considered their role in terms of communication and awareness raising, by sharing alerts, showing the efforts of the food industry and building consumer trust.

### 3.3. Insights from the Interactive Workshop

During the workshop, stakeholders were presented with an overview of the main results of the previous rounds. The four KSFs were presented, and stakeholders were invited to debate a number of results and questions during four parallel working group sessions, which were moderated using a common discussion guide. The discussion guide was developed and structured as indicated in Figure 4, covering the four possible KSFs for an FI-ISS and related discussion points to guide the discussion. Reflection with the four moderators after the sessions showed that the four different groups used the guide in various ways, from very directly following the questions to a looser approach where the stakeholders diverged from the suggested topics. Relevant statements that were made during the sessions are summarized in the discussion grid in Table 3. 

## 4. Conclusions 

Dealing with complex food integrity issues requires a multi-dimensional approach. Besides the development of innovative analytical food process control methods, systems and practices, data and information sharing between actors in the food supply chain could help to facilitate the detection and prevention of food integrity issues. This study has demonstrated positive attitudes towards an FI-ISS among food industry stakeholders in the European food supply chain. Stakeholders are convinced of its benefits for detecting issues more rapidly and inexpensively, and for preventing the occurrence of issues. They believe that sharing information might magnify their own efforts, increase trust and improve the image of the food sector as a whole. Nonetheless, they are concerned about the increase in workload and the cost of such a system. An industry stakeholder’s perception of the advantages of an FI-ISS is a predictor of their intention to join an FI-ISS. Though perceived disadvantages are negatively correlated with perceived advantages, this does not emerge as a significant determinant of intention to join. The perception of the risk of food integrity issues for their organization is not a significant predictor of their intention to join an FI-ISS. Importantly, food industry actors remain somewhat reluctant to trust the information that other actors might share, and they are doubtful about the reaction when a food integrity issue is detected. Medium-sized companies perceive the difficulties regarding the detection of food integrity issues as worse compared to small or larger companies, but still have a lower intention to join an FI-ISS. This empirical finding deserves further attention in future research.

Exposing a broader group of stakeholders to the results of the first round provided additional practical insights and led to the identification of four key success factors for an FI-ISS. Our study shows that the trusted third party that will manage an FI-ISS is preferably a new organization. However, a successful FI-ISS should also involve food safety authorities, albeit in an advisory role rather than a management one. As a third success factor, the study shows that the majority of stakeholders consider an FI-ISS to only be promising if data confidentiality is guaranteed by the data infrastructure. Our study showed that, in spite of their enthusiasm, most stakeholders are skeptical about the ways in which their information could be protected. The fourth key factor for the success of an FI-ISS is the participation of all actors in the food supply chain, including consumer organizations and retailers. 

This stakeholder study faces limitations owing to its relatively small and self-selected sample of stakeholders. There may be selection bias as a result of higher involvement with food integrity issues among the study participants. Stakeholders’ positive attitudes may, therefore, represent a best-case scenario, but are nevertheless encouraging for the development of an FI-ISS. The findings apply within the boundaries of the sample and generalization to the broader population of companies may be speculative. Insights on the barriers that might be encountered can be helpful for the food industry, food safety authorities and the science community in their efforts to ensure future food integrity, and eventually develop an effective FI-ISS.

Relatively high dropout levels of participants were encountered during the first round and between the consecutive rounds of the present study. Future studies might limit dropouts through stimulating the motivation and involvement of participants, e.g., by providing them with more detailed feedback and concrete benefits from the study findings. Another recommendation is to reduce the time lag between consecutive waves of data collection to maintain momentum and interest in the study topic. 

As the development of an FI-ISS evolves, future socio-economic research could focus on monitoring the adoption process and collecting feedback about the experiences of food supply chain stakeholders. Eventually, cost-benefit analysis might be relevant to perform, and thus insight would be provided into what can be gained from the establishment and use of an FI-ISS. Additionally, the integration or linking of an FI-ISS to existing, private or public, traceability or alert systems requires further research. 

## Figures and Tables

**Table 1:** Distribution of participating stakeholders in the three rounds of the stakeholder study.

Type of Stakeholder	*n*	%
**First round**		
Food industry	143	
Large (>250 employees)	60	42.0
Medium-sized (<250 employees)	22	15.4
Small (<50 employees)	21	14.7
Micro (<10 employees)	8	5.6
Not known	32	22.4
**Second round**		
Total number of participants	61	
Food industry	30	49.2
Research	10	16.4
Service to the food industry	9	14.8
Food safety authority	5	8.2
Law enforcement	3	4.9
Consumer organization	2	3.3
Other (e.g., consultants)	2	3.3
**Third round**		
Total number of participants	37	
Research	19	51.3
Food safety authority	8	21.6
Food industry	4	10.8
Government	2	5.4
Other (e.g., consultants)	4	10.8

**Table 2:** Stepwise multiple regression: determinants of intention to join a food integrity information sharing system (FI-ISS) (*n* = 111).

Variables Entered	b	SE	β	t	*p*
*Intention*					
Constant	2.352	0.459		5.125	<0.001
Perceived advantages	0.393	0.115	0.304	3.412	0.001
Medium size	−0.480	0.158	−0.270	−3.028	0.003

b: unstandardized coefficient estimate; SE: standard error; β: standardized coefficient estimate; Model goodness-of-fit: Adjusted *R*^2^ = 13.5%.

**Table 3:** Summary of points raised by the stakeholders during the working group sessions in round 3 (May 2018).

Key Success Factors	Discussion Points
**1. A new organization should manage an FI-ISS** - Consensus in one of the groups about giving the task to a new organization - Some stakeholders prefer a new organization but not a new system, rather a system of systems, in a skeleton architecture or an umbrella - A technology company with the expertise and the know-how under a service contract could execute the technology component, with, on top of that, a public–private partnership for governance	**Should a new organization be private or public?** - Consensus in one of the groups that coordination and communication must be done by a public organization at EU level—funded by EU Commission—but a technical partner should be in charge of data management and data architecture - We expect higher consumer trust in a public organization compared to a private organization - Communication and collaboration between different (national, EU and global) organizations will be crucial - We need to take into account both consumer trust and industry trust, when setting up a system - Impartiality and conflict of interest are important when choosing a trusted third party
	**Who should take the initiative? Who should fund the system?** - The EU Commission should fund the initiative - One group found it unclear who should fund the new organization - Another group agrees that the initiative should be authority-driven
**2. Data and information confidentiality needs to be guaranteed** - Industrial actors can only be in favor of sharing data anonymously, as they are concerned for the possible economic loss - Industry actors are concerned about trust among the general population - Suggestion to share data on different levels, and adjust anonymity to the type of data (early indicators vs outcome indicators)	**Is it feasible to share in order to create a system, guaranteeing that nobody has access to the raw data that are shared?** - Focus should be on the sharing of meaningful data - Full anonymity is not possible: in case of an issue, the involved actors need to be able to be identified - There is a need to share metadata
	**Is the sensitivity of data on volumes and transactions a problem?** - Very low trust between industry actors, according to non-industry actors - Food integrity should be non-competitive, to try to take away some of the pressures of sharing data
	**Other** - At which point do authorities need to be informed of a food integrity issue? - Many data still on paper—need for a shift to machine readable data - By only sharing one-up and one-down, stove pipes are created. This is too restrictive
**3. Food Safety Authorities (FSAs) need to be involved in the FI-ISS** - Part of the issue is that FSAs lack the skillset, for example investigation skills	**Should FSAs have access to data and information?** - Currently it is a game of hide and seek between industry and authorities—industry is not willing to share all data with authorities - Authorities are bound legally
	**Uncertainty about reaction of FSAs in case of issues** - Worry confirmed: when will authorities be informed, and which information will they receive?
**4. All actors in the supply chain need to be in the system** - All European countries need to be involved - Inclusion of regulatory bodies, retail, NGOs - The question remains of whether actors with bad intentions will join?	**Should participation be mandatory?** - Ideally the system should be mandatory but stakeholders doubt that this is feasible - A mandatory system is not possible
	**Accessible for small and medium-sized enterprises (SMEs)?** - Small companies might be frightened to share data - Need for an incentive to convince all partners of the benefits of sharing data - Resource issue for very small companies—involve cooperatives or sector organizations to support SMEs
